# Time until tuberculosis recurrence and associated factors in Brazil: a population-based retrospective cohort study using a linked database

**DOI:** 10.1590/1980-549720240016

**Published:** 2024-04-19

**Authors:** Daniele Maria Pelissari, Lucas Vinícius de Lima, Gabriel Pavinati, Gabriela Tavares Magnabosco, José Nildo de Barros Silva, Patricia Bartholomay, Fernanda Dockhorn Costa Johansen

**Affiliations:** I Ministério da Saúde, Coordenação-Geral de Vigilância da Tuberculose, Endemic Mycoses and Non-Tuberculous Mycobacteria – Brasília (DF), Brazil.; II Universidade Estadual de Maringá, Postgraduate Nursing Program – Maringá (PR), Brazil.; III Ministério da Saúde, Department of HIV/Aids, Tuberculosis, Viral Hepatitis and Sexually Transmitted Infections – Brasília (DF), Brazil.

**Keywords:** Tuberculosis, Recurrence, Cohort studies, Regression analysis, Risk factors, Protective factors, Tuberculose, Recidiva, Estudos de coortes, Análise de regressão, Fatores de risco, Fatores de proteção

## Abstract

**Objective:**

To calculate the rate of tuberculosis recurrence, estimate its average time until recurrence, and identify factors associated with recurrence in Brazil.

**Methods:**

Retrospective cohort study with a linked database from the Notifiable Diseases Information System. The study included individuals diagnosed with tuberculosis in 2015, focusing on those who experienced their first recurrence within 6.5 years. We estimated the relative risk (RR) and its 95% confidence interval (95%CI), as well as the population attributable fraction (PAF) or the population preventable fraction (PPF) of associated factors.

**Results:**

Within a 6.5-year period, 3,253 individuals (6.5%) experienced tuberculosis recurrence, with a median time of 2.2 years. Positively associated factors included: male sex (RR: 1.4; 95%CI 1.3–1.5; PAF: 22.9%), age 30 to 59 years (RR: 3.0; 95%CI 1.6–5.7; PAF: 36.0%), black race (RR: 1.3; 95%CI 1.2–1.5; PAF: 3.5%), mixed race (RR: 1.3; 95%CI 1.2–1.4; PAF: 10.6%), deprivation of liberty (RR: 1.9; 95%CI 1.7–2.1; PAF: 9.1%), pulmonary/mixed clinical form (RR: 1.7; 95%CI 1.4–1.9; PAF: 37.1%), acquired immunodeficiency syndrome diagnosis (RR: 1.8; 95%CI 1.5–1.9; PAF: 4.3%), and alcohol use (RR: 1.2; 95%CI 1.1–1.3; PAF: 2.9%). Negatively associated factors were: 12 or more years of schooling (RR: 0.5; 95%CI 0.4–0.6; PPF: 3.3%) and supervised treatment (RR: 0.9; 95%CI 0.8–0.9; PPF: 4.4%).

**Conclusion:**

This study revealed high tuberculosis recurrence rates in Brazil, influenced by sociodemographic, compartmental, and social factors, both positively and negatively impacting disease recurrence.

## RESUMO

**Objetivo:**

Calcular a taxa de recorrência de tuberculose, estimar seu tempo médio e identificar seus fatores associados no Brasil.

**Métodos:**

Estudo de coorte retrospectiva com dados de *linkage* do Sistema de Informação de Agravos de Notificação. Incluímos pessoas diagnosticadas com tuberculose em 2015, com foco naquelas que tiveram sua primeira recorrência em 6,5 anos. Estimamos o risco relativo (RR) e seus intervalos de confiança de 95% (IC95%), assim como a fração atribuível populacional (FAP) ou a fração prevenível populacional (FPP) dos fatores associados.

**Resultados:**

No período de 6,5 anos, 3.253 indivíduos (6,5%) tiveram recorrência de tuberculose, com tempo médio de 2,2 anos. Fatores positivamente associados incluíram: sexo masculino (RR: 1,4; IC95% 1,3–1,5; FAP: 22,9%), idade de 30 a 59 anos (RR: 3,0; IC95% 1,6–5,7; FAP: 36,0%), raça/cor preta (RR: 1,3; IC95% 1,2–1,5; FAP: 3,5%) ou raça/cor parda (RR: 1,3; IC95% 1,2–1,4; FAP: 10,6%), privação de liberdade (RR: 1,9; IC95% 1,7–2,1; FAP: 9,1%), forma clínica pulmonar/mista (RR: 1,7; IC95% 1,4–1,9; FAP: 37,1%), diagnóstico de síndrome da imunodeficiência adquirida (RR: 1,8; IC95% 1,5–1,9; FAP: 4,3%) e uso de álcool (RR: 1,2; IC95% 1,1–1,3; FAP: 2,9%). Fatores negativamente associados foram: 12 ou mais anos de estudo (RR: 0,5; IC95% 0,4–0,6; FPP: 3,3%) e tratamento supervisionado (RR: 0,9; IC95% 0,8–0,9; FPP: 4,4%).

**Conclusão:**

Revelamos taxas elevadas de recorrência de tuberculose no Brasil, com fatores sociodemográficos, comportamentais e sociais influenciando na recorrência da doença.

## INTRODUCTION

Tuberculosis (TB) remains a priority for the World Health Organization (WHO) due to its significant impact on morbidity and mortality rates, especially in developing countries. In 2021, the estimated worldwide number of people with TB reached 10.6 million, and 1.6 million succumbed to the disease^1^. In pursuit of ending TB as a public health problem by 2035, the WHO identified 30 priority countries for control programs, including Brazil^1^.

In 2022, the TB incidence rate in Brazil was 37.4 cases per 100,000 inhabitants, with a mortality rate of 2.61 deaths per 100,000 inhabitants^2^. The country faces numerous challenges in controlling the disease, such as healthcare resource inequalities, low education levels, income and occupation disparities, high population density in vulnerable socioeconomic territories, poor living conditions, and elevated loss to follow-up rates that sustain the transmission chain^3,4^.

Among the complicating factors hindering progress towards the End TB Strategy, cases of re-treatment, whether due to relapse or reinfection, are particularly noteworthy. TB recurrence is defined as an episode of TB that occurs after the completion of anti-TB treatment^5^. Following successful treatment, some individuals may experience a new occurrence of the disease due to either endogenous reactivation of the initial infection or acquisition of a new exogenous infection^5^.

Between 2015 and 2022, data from the Notifiable Diseases Information System (*Sistema de Informação de Agravos de Notificação* – SINAN) reported over 54,000 cases of TB re-treatment in Brazil due to both reactivation and reinfection^6^. There was a percentage increase of 8.6% when comparing the years 2015 and 2019 (coronavirus disease [COVID-19] pre-pandemic period), and a 2.3% increase when comparing the years 2015 and 2022 (pandemic period)^6^.

TB recurrence can be attributed to individual factors such as male sex, 60 years of age or more, comorbidities (diabetes, renal failure, and systemic diseases, particularly human immunodeficiency virus [HIV] infection), low income, and underweight. Additionally, programmatic and epidemiological factors, such as high-incidence TB settings, treatment failure, and non-utilization of directly observed treatment (DOT), can be positively associated with TB recurrence^7-9^.

Therefore, understanding the risk factors for TB recurrence is crucial for comprehending the epidemiological scenario and accelerating progress towards the elimination of the disease in Brazil by 2035. Using data from SINAN, we identified episodes of TB treatment in the same individual over a 6.5-year period. From this, we calculated the recurrence rate, estimated the average time until recurrence, and identified factors associated with TB recurrence in Brazil.

## METHODS

### Study design and setting

We conducted a population-based retrospective cohort study following the guidelines of the Reporting of Studies Conducted using Observational Routinely-Collected Health Data (RECORD). Brazil, situated in South America, had a population of 214 million in 2021. As an upper-middle-income country characterized by significant social and economic inequality, its gross domestic product (GDP) per capita was R$ 42,247.52 (US$ 7,696.80), and its Gini index was 52.9 in 2021^10^.

### Data source

In Brazil, notification of TB cases is mandatory and recorded in a decentralized surveillance system, facilitating the dynamic diagnosis of events. We obtained data from SINAN for new TB patients diagnosed in 2015. Subsequently, we conducted a search for episodes of TB recurrence in these patients using the complete database of cases notified between January 2015 and May 2022 (6.5 years). For this purpose, we employed a probabilistic record linkage approach with RecLink III^®^ software.

Following the standardization of variables, the linkage process was based on four blocks, involving combinations with the soundex of the person's first and last name, as well as their sex. Additional data, such as the person's name, mother's name, birthdate, state and municipality of residence, and complete address, were also utilized. From this information, we estimated probability scores that indicated the likelihood that two records would belong to the same individual.

### Population

According to the WHO, TB recurrence is defined as individuals who have been treated for TB, declared cured or completed treatment, and are subsequently diagnosed with a new episode of TB, encompassing either reinfection or relapse^11^. However, due to the absence of molecular genotyping data in SINAN, it was not feasible to differentiate between relapse and reinfection in this study. Therefore, both relapse and reinfection cases were collectively referred to as TB recurrence cases.

**Figure 1:**
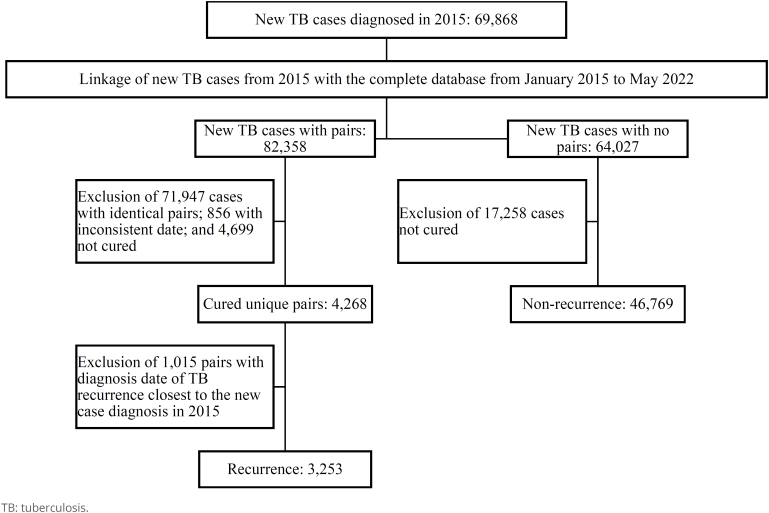
Steps for probabilistic record linkage of tuberculosis new cases diagnosed in 2015, with and without recurrence, using the complete databases of tuberculosis cases in Brazil from January 2015 to May 2022.

We included new TB cases diagnosed in 2015 who had received treatment or had been on anti-TB drugs for less than one month, as well as individuals with an unknown treatment history. Our specific focus was on those who experienced their first recurrence within a 6.5-year period and were successfully matched in the record linkage process. The comparison group consisted of new TB cases diagnosed in 2015 who were declared cured or had completed treatment but were not matched in the linkage (Figure 1).

### Variables

In this study, demographic and socioeconomic variables from the first episode of TB included the following categories: sex (male and female); age (in years); education level (0–8, 9–11, and 12 or more years of schooling); race (white, mixed, black, Asian, and indigenous); beneficiary of a cash transfer program (yes and no); person deprived of liberty (yes and no); homeless person (yes and no); health professional (yes and no); and immigrant population (yes and no).

Regarding the clinical and behavioral variables, we analyzed: clinical form of TB (pulmonary and mixed [i.e., both pulmonary and extrapulmonary forms], and extrapulmonary); diabetes (yes and no); HIV status (positive, negative, acquired immunodeficiency syndrome [AIDS], and unknown); tobacco use (yes and no); alcohol use (yes and no); illicit drug use (yes and no); and DOT (yes and no). Some variables had a subcategory indicating missing data, labeled as "not informed".

As no progressive association with TB recurrence was observed with the variable "age (in years)", we opted to analyze it categorically by age groups (0–4, 5–9, 10–14, 15–19, 20–29, 30–59, and 60 years and older). Regarding DOT, the Brazilian Ministry of Health defines it — at the time this study was developed — as a treatment approach where a trained healthcare worker directly observes the patient swallowing the medication at least three times a week throughout the entire treatment period.

### Data analysis

We calculated the recurrence rate as the percentage of recurrent cases in the overall population, multiplied by 100. The cases of TB recurrence and non-recurrence were described using relative frequencies. Time to recurrence was determined by calculating the difference between the end of the first episode and the diagnosis date of the second episode. We estimated the median and mean time, the interquartile range (IQR: 25%–75%), and the standard deviation (SD).

To identify factors associated with TB recurrence, we calculated the relative risk (RR) and its 95% confidence interval (95%CI) using Poisson regression with robust variance in Stata^®^, v. 14. The dependent variable included cases matched in the record linkage (recurrence group) and those not matched (non-recurrence group). For the independent variables, we employed a theoretical and statistical approach to determine the factors included in the models.

Initially, we selected variables from the SINAN dataset that had shown an association with TB and/or TB recurrence in previous studies^5,7,9,12-14^. Among these variables, those with a p-value (p)≤0.20 in the bivariate analysis were included in the multiple models using stepwise backward selection. Subsequently, only variables with p≤0.05 remained in the final model. The regression coefficients were then exponentiated to estimate the adjusted RR (aRR) and its 95%CI.

Variables with more than 10% missing data were incorporated into the model as a distinct subcategory. To address the challenge of missing data and minimize potential biases in the final model, we performed a sensitivity analysis using multiple imputations with the Amelia package in R Studio^®^. This method utilized a bootstrapping and expectation-maximization algorithm to impute missing values in the dataset, employing the missing completely at random (MCAR) approach.

Additionally, we also calculated the population attributable fraction (PAF) using Miettinen's formula to estimate the proportion of recurrence incidence attributed to a positively associated factor (aRR>1.00)^15^. In the case of a negatively associated factor (aRR<1.00), we estimated the population preventable fraction (PPF) using Walter's formula to measure the proportion of recurrence that could be avoided if everyone were exposed to a specific factor^15^.

### Ethical considerations

The record linkage was conducted by the Brazilian Ministry of Health as part of their routine surveillance activities, and the database can be accessed by request on the platform *Fala.BR* website. Since this study did not involve data containing patient identification, it was exempt from submission to the Research Ethics Committee under Brazilian ethical recommendations (Resolution no. 674, dated May 6, 2022, of the Brazilian National Health Council).

## RESULTS

In 2015, a total of 50,022 new cases of TB were declared cured or completed treatment. Among them, 3,253 (6.5%) experienced TB recurrence over a 6.5-year follow-up period. Conversely, 46,796 new TB cases from 2015 did not exhibit a recurrence. The median time to TB recurrence was 2.2 years (IQR: 1.0–3.8), and the mean time was 2.5 years (SD: 1.8).

In specific population groups, both children (aged 5–9 years) and the elderly had a median time to TB recurrence of 1.4 years. Vulnerable populations showed shorter times to TB recurrence, with the following medians: homeless individuals (1.7 years), health professionals (1.6 years), immigrants (1.6 years), people with HIV (1.6 years), and people with AIDS (1.8 years) (Table 1).

**Table 1:** Univariate analysis of demographic, socioeconomic, clinical, and behavioral characteristics of tuberculosis recurrence and non-recurrence cases in Brazil, January 2015 to May 2022.

Variable		TB no recurrence	TB recurrence	Median time
		n (%)	n (%)	Years (IQR)
Sex				
	Female	16,015 (34.2)	716 (22.0)	2.0 (0.0–6.3)
	Male	30,752 (65.8)	2,537 (78.0)	2.2 (0.0–6.6)
	Not informed	2 (0.0)	0 (0.0)	NA
Age group (in years)				
	0 to 4	622 (1.3)	13 (0.4)	4.3 (3.0–4.3)
	5 to 9	321 (0.7)	7 (0.2)	1.4 (2.3–0.5)
	10 to 14	687 (1.5)	24 (0.7)	2.7 (1.3–4.9)
	15 to 19	3,256 (7.0)	194 (6.0)	2.5 (0.1–6.0)
	20 to 29	11,222 (24.0)	870 (26.7)	2.4 (0.0–6.4)
	30 to 59	24,240 (51.8)	1,743 (53.7)	2.1 (0.0–6.5)
	60 and more	6,419 (13.7)	401 (12.3)	1.4 (0.0–6.2)
Race				
	White	16,009 (34.2)	858 (26.4)	2.3 (0.0–6.4)
	Black	5,362 (11.5)	449 (13.8)	2.1 (0.0–6.1)
	Asian	338 (0.7)	27 (0.8)	2.6 (0.7–4.1)
	Mixed	20,990 (44.9)	1,602 (49.2)	2.2 (0.0–6.5)
	Indigenous	644 (1.4)	25 (0.8)	1.7 (0.3–3.4)
	Not informed	3,426 (7.3)	292 (9.0)	NA
Education level (in years)				
	0 to 8	20,620 (44.0)	1,819 (55.9)	2.1 (0.0–6.5)
	9 to 11	11,253 (24.1)	501 (15.4)	2.4 (0.0–6.2)
	12 and more	3,320 (7.1)	94 (2.9)	2.3 (0.1–6.2)
	Not informed	11,576 (24.8)	839 (25.8)	NA
Beneficiary of a cash transfer program				
	No	21,548 (46.1)	1,864 (57.3)	2.1 (0.0–6.4)
	Yes	2,254 (4.8)	182 (5.6)	2.3 (0.0–6.3)
	Not informed	22,967 (49.1)	1,207 (37.1)	NA
Prison population				
	No	38,543 (82.4)	2,274 (69.9)	2.1 (0.0–6.5)
	Yes	3,896 (8.3)	630 (19.4)	2.5 (0.0–6.4)
	Not informed	4,330 (9.3)	349 (10.7)	NA
Homeless population				
	No	41,378 (88.4)	2,766 (85.0)	2.2 (0.0–6.6)
	Yes	634 (1.4)	70 (2.2)	1.7 (0.3–5.9)
	Not informed	4,757 (10.2)	417 (12.8)	NA
Health professional				
	No	41,277 (88.2)	2,820 (86.7)	2.2 (0.0–6.6)
	Yes	680 (1.5)	19 (0.6)	1.6 (0.9–3.8)
	Not informed	4,812 (10.3)	414 (12.7)	NA
Immigrant population				
	No	39,718 (85.0)	2,766 (85.1)	2.2 (0.0–6.6)
	Yes	205 (0.4)	8 (0.2)	1.6 (1.2–2.0)
	Not informed	6,846 (14.6)	479 (14.7)	NA
Clinical form				
	Extrapulmonary	6,473 (13.8)	225 (6.9)	2.0 (0.0–6.0)
	Pulmonary/mixed	40,296 (86.2)	3,028 (93.1)	2.2 (0.0–6.6)
	Not informed	0 (0.0)	0 (0.0)	NA
HIV status				
	Negative	35,658 (76.2)	2,308 (70.9)	2.3 (0.0–6.5)
	Positive	385 (0.8)	41 (1.3)	1.6 (0.3–5.5)
	AIDS	2,744 (5.9)	320 (9.8)	1.8 (0.0–6.0)
	Unknown	7,982 (17.1)	584 (18.0)	2.2 (0.0–6.2)
Diabetes				
	No	40,456 (86.5)	2,645 (81.3)	2.2 (0.0–6.5)
	Yes	3,441 (7.4)	236 (7.3)	2.1 (0.0–5.8)
	Not informed	2,872 (6.1)	372 (11.4)	NA
Tobacco use				
	No	35,747 (76.4)	2,153 (66.2)	2.2 (0.0–6.5)
	Yes	7,287 (15.6)	681 (20.9)	2.2 (0.0–6.4)
	Not informed	3,735 (8.0)	419 (12.9)	NA
Alcohol use				
	No	37,901 (81.0)	2,318 (71.2)	2.2 (0.0–6.5)
	Yes	6,126 (13.1)	607 (18.7)	2.1 (0.0–6.3)
	Not informed	2,742 (5.9)	328 (10.1)	NA
Illicit drug use				
	No	39,175 (83.8)	2,415 (74.2)	2.1 (0.0–6.6)
	Yes	3,651 (7.8)	399 (12.3)	2.4 (0.0–6.0)
	Not informed	3,943 (8.4)	439 (13.5)	NA
DOT				
	No	16,827 (36.0)	1,149 (35.3)	2.1 (0.0–6.4)
	Yes	19,532 (41.7)	1,299 (40.0)	2.2 (0.0–6.3)
	Not informed	10,410 (22.3)	805 (24.7)	NA

TB: tuberculosis; IQR: interquartile range (25%–75%); HIV: human immunodeficiency virus; DOT: directly observed treatment; AIDS: acquired immunodeficiency syndrome; NA: not applicable.

Among cases of TB recurrence, a higher proportion were male (78.0%) compared to non-recurrence cases (65.8%). Black (13.8%) and mixed (49.2%) individuals were more prevalent among TB recurrence cases than in the comparison group (black: 11.5%; mixed: 44.9%). Cases without recurrence had more years of schooling compared to TB recurrence cases (Table 1).

Prison and homeless populations were more prevalent among TB recurrence cases (prison: 19.4%; homeless: 2.2%) compared to those without a recurrence (prison: 8.3%; homeless: 1.4%). In contrast, health professionals (0.6% among TB recurrence and 1.5% in the comparison group) and the immigrant population (0.2% among TB recurrence and 0.4% in the comparison group) were less prevalent (Table 1).

The pulmonary/mixed clinical form of TB was more frequent in the recurrence group (93.1%). Additionally, TB recurrence cases had a higher proportion of individuals with AIDS (9.8%) and a higher prevalence of tobacco use (20.9%), alcohol consumption (18.7%), and illicit drug use (12.3%). Finally, the proportion of individuals with TB recurrence who underwent DOT was slightly lower (40.0%) than non-recurrence cases (Table 1).

Except for three variables (diabetes, beneficiary of a cash transfer program, and immigrant population), all the other independent variables showed significant association with TB recurrence in the bivariate analysis (p≤0.20) (Table 2). Subsequently, eleven variables remained associated with TB recurrence in the final model of the multivariate analysis (p≤0.05) (Table 3).

**Table 2:** Bivariate analysis of demographic, socioeconomic, clinical, and behavioral factors associated with tuberculosis recurrence in Brazil, January 2015 to May 2022.

Variable		uRR (95%CI)
Sex*		
	Female	Reference
	Male	1.8 (1.6–1.9)†
Age group (in years)*		
	0 to 4	Reference
	5 to 9	1.0 (0.4–2.6)
	10 to 14	1.6 (0.8–3.2)
	15 to 19	2.7 (1.6–4.8)†
	20 to 29	3.5 (2.0–6.0)†
	30 to 59	3.3 (1.9–5.6)†
60 and more		2.9 (1.7–5.0)†
	Race*	
	White	Reference
	Black	1.5 (1.4–1.7)†
	Asian	1.5 (1.0–2.1)†
	Mixed	1.4 (1.3–1.5)†
	Indigenous	0.7 (0.5–1.1)
Education level (in years)*		
	0 to 8	Reference
	9 to 11	0.5 (0.5–0.6)†
	12 and more	0.3 (0.3–0.4)†
	Not informed	0.8 (0.8–0.9)†
Beneficiary of a cash transfer program		
	No	Reference
	Yes	0.9 (0.8–1.1)
	Not informed	0.6 (0.6–0.7)†
Prison population*		
	No	Reference
	Yes	2.5 (2.3–2.7)†
	Not informed	1.3 (1.2–1.5)†
Homeless population*		
	No	Reference
	Yes	1.6 (1.3–2.0)†
	Not informed	1.3 (1.2–1.4)†
Health professional*		
	No	Reference
	Yes	0.4 (0.3–0.7)†
	Not informed	1.2 (1.1–1.4)†
Immigrant population		
	No	Reference
	Yes	0.6 (0.3–1.1)
	Not informed	1.0 (0.9–1.1)
Clinical form*		
	Extrapulmonary	Reference
	Pulmonary/mixed	2.1 (1.8–2.4)†
HIV status*		
	Negative	Reference
	Positive	1.6 (1.2–2.1)†
	AIDS	1.7 (1.5–1.9)†
	Unknown	1.1 (1.0–1.2)
Diabetes		
	No	Reference
	Yes	1.0 (0.9–1.2)
	Not informed	1.9 (1.7–2.1)†
Tobacco use*		
	No	Reference
	Yes	1.5 (1.4–1.6)†
	Not informed	1.8 (1.6–2.0)†
Alcohol use*		
	No	Reference
	Yes	1.6 (1.4–1.7)†
	Not informed	1.9 (1.7–2.1)†
Illicit drug use*		
	No	Reference
	Yes	1.7 (1.5–1.9)†
	Not informed	1.7 (1.6–1.9)†
DOT*		
	No	Reference
	Yes	1.0 (0.9–1.1)
	Not informed	1.1 (1.0–1.2)

uRR: unadjusted relative risk; 95%CI: 95% confidence interval (lower bound–upper bound); HIV: human immunodeficiency virus; DOT: directly observed treatment; AIDS: acquired immunodeficiency syndrome;

* p≤0.20;

† p≤0.05.

**Table 3:** Multivariate analysis of demographic, socioeconomic, clinical, and behavioral factors associated with tuberculosis recurrence, proportion rate, and population attributable and preventable fraction in Brazil, January 2015 to May 2022.

Variable		Rate	aRR (95%CI)	PAF/PPF (95%CI)
Sex				
	Female	22.0	Reference	Reference
	Male	78.0	1.4 (1.3–1.5)*	22.9 (18.1–27.8)*
Age group (in years)				
	0 to 4	0.4	Reference	Reference
	5 to 9	0.2	1.4 (0.6–3.8)	0.1 (-0.1–0.2)
	10 to 14	0.7	2.0 (1.0–4.1)	0.4 (0.1–0.6)
	15 to 19	6.0	3.2 (1.7–6.1)*	4.1 (3.0–5.3)*
	20 to 29	26.7	3.1 (1.7–5.8)*	18.1 (12.8–23.5)*
	30 to 59	53.6	3.0 (1.6–5.7)*	36.0 (25.1–46.9)*
	60 and more	12.3	2.9 (1.6–5.5)*	8.1 (5.5–10.7)*
Race				
	White	26.4	Reference	Reference
	Black	13.8	1.3 (1.2–1.5)*	3.5 (2.3–4.6)*
	Asian	0.8	1.4 (1.0–2.1)	0.3 (0.0–0.5)
	Mixed	49.2	1.3 (1.2–1.4)*	10.6 (7.5–13.8)*
	Indigenous	0.8	0.9 (0.6–1.3)	-0.1 (-0.5–0.2)
Education level (in years)				
	0 to 8	55.9	Reference	Reference
	9 to 11	15.4	0.6 (0.5–0.6)*	10.1 (7.8–12.2)*
	12 and more	2.9	0.5 (0.4–0.6)*	3.3 (2.1–4.6)*
	Not informed	25.8	0.8 (0.8–0.9)*	5.2 (2.6–7.6)*
Prison population				
	No	69.9	Reference	Reference
	Yes	19.4	1.9 (1.7–2.1)*	9.1 (8.1–10.1)*
	Not informed	10.7	1.1 (1.0–1.3)	1.2 (0.1–2.4)
Clinical form				
	Extrapulmonary	6.9	Reference	Reference
	Pulmonary/mixed	93.1	1.7 (1.4–1.9)*	37.1 (29.4–44.8)*
HIV status				
	Negative	70.9	Reference	Reference
	Positive	1.3	1.5 (1.1–2.0)*	0.4 (0.2–0.7)*
	AIDS	9.8	1.8 (1.6–2.0)*	4.3 (3.6–4.9)*
	Unknown	18.0	1.0 (0.9–1.1)	0.7 (-1.0–2.3)
Tobacco use				
	No	66.2	Reference	Reference
	Yes	20.9	1.2 (1.0–1.3)	2.7 (1.0–4.5)
	Not informed	12.9	1.2 (0.9–1.5)	2.0 (-0.6–4.6)
Alcohol use				
	No	71.3	Reference	Reference
	Yes	18.7	1.2 (1.1–1.3)*	2.9 (1.3–4.5)*
	Not informed	10.1	1.1 (0.9–1.3)	1.1 (-0.5–2.7)
Illicit drug use				
	No	74.2	Reference	Reference
	Yes	12.3	1.1 (1.0–1.3)	1.4 (0.1–2.6)
	Not informed	13.5	1.1 (0.9–1.4)	1.0 (-1.9–4.0)
DOT				
	No	35.3	Reference	Reference
	Yes	39.9	0.9 (0.8–0.9)*	4.4 (1.1–7.6)*
	Not informed	24.7	1.0 (0.9–1.1)	0.3 (-2.0–2.5)

aRR: adjusted relative risk; 95%CI: 95% confidence interval (lower bound–upper bound); PAF: population attributable fraction; PPF: population preventable fraction; HIV: human immunodeficiency virus; DOT: directly observed treatment; AIDS: acquired immunodeficiency syndrome;

* p≤0.05.

The identified positively associated factors with TB recurrence were: male sex; age of 15 years old or more; black or mixed race; prison population; pulmonary/mixed form of TB; HIV or AIDS coinfection; and alcohol use. The negatively associated factors with TB recurrence were: nine years or more of schooling and supervised treatment (DOT) (Table 3). In the sensitivity analysis, most of these factors remained associated and had similar RR values (Table 4).

**Table 4:** Bivariate and multivariate sensitivity analysis of demographic, socioeconomic, clinical, and behavioral factors associated with tuberculosis recurrence in the final model of the principal analysis in Brazil, January 2015 to May 2022.

Variable		uRR (95%CI)	aRR (95%CI)
Sex			
	Female	Reference	Reference
	Male	1.8 (1.6–1.9)*	1.4 (1.3–1.5)*
Age group (in years)			
	0 to 4	Reference	Reference
	5 to 9	0.8 (0.3–2.1)	0.8 (0.3–2.2)
	10 to 14	1.6 (0.8–3.0)	1.4 (0.7–3.1)
	15 to 19	2.6 (1.5–4.4)*	2.3 (1.2–4.4)*
	20 to 29	3.3 (1.9–5.5)*	2.3 (1.2–4.4)*
	30 to 59	3.0 (1.8–5.1)*	2.2 (1.1–4.2)*
	60 and more	2.7 (1.6–4.5)*	2.1 (1.1–4.0)*
Race			
	White	Reference	Reference
	Black	1.4 (1.3–1.6)*	1.3 (1.1–1.4)*
	Asian	1.4 (1.0–1.9)	1.3 (1.0–1.8)
	Mixed	1.3 (1.2–1.5)*	1.2 (1.2–1.3)*
	Indigenous	1.0 (0.7–1.3)	1.1 (0.8–1.4)
Education level (in years)			
	0 to 8	Reference	Reference
	9 to 11	0.6 (0.6–0.7)*	0.7 (0.6–0.7)*
	12 and more	0.5 (0.4–0.6)*	0.6 (0.5–0.7)*
Prison population			
	No	Reference	Reference
	Yes	2.2 (2.1–2.4)*	1.8 (1.7–2.0)*
Clinical form			
	Extrapulmonary	Reference	Reference
	Pulmonary/mixed	2.1 (1.8–2.4)*	1.7 (1.5–2.0)*
HIV status			
	Negative	Reference	Reference
	Positive	1.4 (1.2–1.8)*	1.4 (1.1–1.7)*
	AIDS	1.5 (1.4–1.7)*	1.6 (1.4–1.7)*
Tobacco use			
	No	Reference	Reference
	Yes	1.5 (1.4–1.6)*	1.2 (1.1–1.3)*
Alcohol use			
	No	Reference	Reference
	Yes	1.5 (1.4–1.6)*	1.2 (1.1–1.3)*
Illicit drug use			
	No	Reference	Reference
	Yes	1.6 (1.5–1.8)*	1.1 (1.0–1.2)
DOT			
	No	Reference	Reference
	Yes	1.0 (0.9–1.1)	0.9 (0.9–1.0)

uRR: unadjusted relative risk; 95%CI: 95% confidence interval (lower bound–upper bound); aRR: adjusted relative risk; HIV: human immunodeficiency virus; DOT: directly observed treatment; AIDS: acquired immunodeficiency syndrome;

* p≤0.05.

The PAF indicated that 22.9% of TB recurrence cases were attributed to men, while the age group of 30–59 years contributed to 36.0% of cases. Black and mixed races accounted for 3.5% and 10.6% of TB recurrence cases, respectively. The prison population had a PAF of 9.1%, and the pulmonary/mixed clinical form accounted for 37.1% of cases. Individuals with AIDS had a PAF of 4.3%. Among the substances, alcohol stood out with 2.9% (Table 3).

The fraction of TB recurrence cases that could be preventable due to protective factors is presented in Table 3. We found that having 9–11 years of education could prevent 10.1% of TB recurrence cases. Additionally, 3.3% of recurrence cases could be avoided if individuals had 12 or more years of schooling. Expanding DOT to all cases could prevent 4.4% of TB recurrence cases.

## DISCUSSION

The recurrence rate over a 6.5-year period for TB cases with successful treatment in the first episode was 6.5%. Social vulnerabilities, such as belonging to mixed/black races, having a low level of education, and being in prison, were identified as positively associated factors. The pulmonary/mixed clinical form of TB and HIV/AIDS coinfection showed an increased risk, as did alcohol use. Undergoing DOT and having over nine years of schooling were found to be negatively associated factors.

The recurrence rate results in our study align with findings from a meta-analysis conducted in resource-limited and high TB incidence countries, estimating a relapse rate of 5.6% within 18 to 24 months of follow-up after a standard 6-month regimen^16^. Similarly, a cohort study in Cape Town, South Africa, reported an 8.0% recurrence rate over a 13-year follow-up period^17^. In a national study in Korea, utilizing a linked routine surveillance database, a 5.0-year relapse rate of 9.7% was reported^18^.

However, a prospective longitudinal study in Jiangxi province, China, observed a higher recurrence rate (15.2%) among patients over 14 years old followed up for seven years^12^. Factors such as high-burden settings favoring reinfection of cured patients, the quality of treatment and follow-up influencing the reactivation of TB, and methodological variations in the studies (e.g., recurrence definition, follow-up period, study design, and population) may account for the differences in recurrence rates.

Our data did not allow us to distinguish between relapse and reinfection cases. However, we can hypothesize which one occurred. A study conducted in Cape Town, a setting with a higher TB burden than Brazil, indicated that relapse occurred shortly after treatment completion, while reinfection became dominant after one year and accounted for at least half of the recurrent cases^18^. Considering this and our median time of 2.2 years, it is plausible that most recurrence TB cases in Brazil were due to reinfection.

Male sex is an established positively associated factor with TB recurrence^7,9,12^. Our data show a significant rate of recurrence attributed to males and individuals aged over 15 years old. Notably, men are more likely to engage in behaviors, such as difficulties in identifying their health demands and the non-adoption of protective practices, that can lead to unfavorable treatment outcomes (e.g., loss to follow-up)^4,19,20^. This could explain the higher proportion of TB recurrence in this group.

The elderly population is known to be more susceptible to a weakened immune system, particularly due to underlying diseases that cause immunosuppression. Additionally, they often experience adverse drug reactions resulting from interactions between anti-TB medications and other drugs^21,22^. This situation can contribute to the reactivation of TB, consistent with the short median time to recurrence that we observed in this population (1.4 years; IQR: 0.0–6.2).

Studies that analyzed income with different measures have consistently found an association with TB recurrence. A retrospective cohort study in Henan province, China, demonstrated a strong link between low annual household income and TB recurrence^23^. A population-based cohort study in South Korea also reported a similar association^14^. Additionally, a research conducted in Blantyre, Malawi, revealed that individuals affected by TB remained economically vulnerable even after completing treatment^24^.

Although we did not directly measure income, schooling and race serve as proxies for this factor. We found a positive association of TB recurrence with a low level of education and mixed race. Historically, the black/mixed population in Brazil has had lower levels of education and income^25^. Consequently, individuals in these groups have a lower probability of accessing healthcare services^26^ and experience higher incidence rates^27,28^. These factors could contribute to TB reactivation and/or reinfection.

Studies in prisons, including in Brazil, have revealed high rates of TB due to overcrowding, poor environmental conditions, and delays in diagnosis^29,30^. In our study, the median time to TB recurrence was 2.5 years (IQR: 0.0–6.4), suggesting that reinfection may be the underlying mechanism. Although we found that 9.1% of recurrences were attributable to being in prison, it is noteworthy that TB treatment can be facilitated due to confinement in an apparently controlled environment^4^.

We observed a higher risk of recurrence in cases with the pulmonary/mixed TB form. Given the elevated prevalence of this clinical form among recurrent cases, the PAF was also particularly high at 37.1%. A previous study indicated that in areas with a high TB incidence, the proportion of reinfections increases, likely due to new exposures^5^. Hence, it can be inferred that TB recurrence in the pulmonary form may be associated with greater exposure and reinfection.

We identified an association between alcohol use and recurrent TB, consistent with previous studies^12,31^ that also documented this association. A study in the Democratic Republic of Congo found that alcoholism increased the risk of TB recurrence by 3.9 times^31^. In China, a study reported a 2.5 times higher risk of TB recurrence among tobacco users, prompting the authors to recommend expanding counseling strategies to address substance use and closely monitoring patients after TB treatment^12^.

In settings with a high burden of TB, reinfection may explain the elevated rates of recurrence among individuals with HIV^17^. However, our study yielded different results. As expected^32^, the HIV-positive population was associated with recurrence, but they presented a shorter time to re-treatment, suggesting that the mechanism of recurrence in these individuals in Brazil is endogenous reactivation. Further studies are needed to explore the impact of TB-HIV coinfection and the quality of patient follow-up.

Undergoing supervised treatment was a protective factor against TB recurrence in our study. Furthermore, we found that 4.4% (95%CI 1.1–7.6) of recurrence cases could be prevented if DOT were expanded to all affected individuals in their first episode. However, among the recurrence cases, 60.8% of them did not receive this treatment strategy, underscoring the potential of enhancing the qualification of DOT to prevent re-treatment in TB cases in Brazil.

These findings reinforce other Brazilian studies that have identified weaknesses in the implementation of decentralized DOT for TB in primary health care, primarily due to structural and operational challenges in these services^33,34^. It is important to mention that DOT should be implemented for all TB cases. However, primary health care in Brazil still faces issues with inadequate structures and work processes^35^, which may explain the low implementation rate of the strategy.

Our study has certain limitations. We acknowledge the possibility of underreporting since data were extracted from SINAN, which relies on notifications from health services that may not consistently report all cases. We also recognize the absence of certain variables that were associated with recurrence (e.g., malnutrition and low body weight)^13^ as a limitation. Furthermore, the lack of genotyping data in our study prevented us from distinguishing between relapse and reinfection.

Finally, we emphasize a limitation in our study related to assuming missing completely at random when utilizing the Amelia package for the model that underwent multiple data imputations. However, it is noteworthy that this does not invalidate our obtained results. The associations derived from both multivariate models, with and without imputation, remained consistent with what was observed in the literature for certain specific outcomes in TB treatment^23,24,33^.

Overall, national data from our cohort study strongly suggest that the majority of recurrences during the 6.5-year observation period were likely due to reinfection. However, considering the shorter time to recurrence for specific population groups such as children, the elderly, and people living with HIV, the underlying mechanism appeared to be a relapse of the initial episode. These findings emphasize the need to improve clinical management practices and public policies for TB control in Brazil.

We identified social vulnerabilities such as mixed/black race, low level of education, and being in prison as risk factors for TB recurrence. In terms of clinical aspects, the pulmonary/mixed clinical form of TB and HIV/AIDS coinfection demonstrated a strong association with increased risk, as did alcohol use. Conversely, undergoing supervised treatment and having over nine years of schooling were identified as protective factors against TB recurrence.

In light of these findings, preventing TB re-treatment cases is crucial through the implementation of practices aimed at monitoring and providing follow-up care to individuals being treated for TB or those who have completed treatment, especially for groups with higher rates of recurrence in our study. Thus, we underscore the importance of person-centered care, including strategies such as DOT and individualized treatment plans, which can significantly contribute to the effectiveness of TB control programs.
